# Fatigue Analysis of the Microturbine Rotor Disc Made of 7075 Aluminium Alloy Using a New Hybrid Calculation Method

**DOI:** 10.3390/ma15030834

**Published:** 2022-01-22

**Authors:** Paweł Zych, Grzegorz Żywica

**Affiliations:** Institute of Fluid Flow Machinery, Polish Academy of Sciences, Fiszera 14, 80-231 Gdansk, Poland; pzych@imp.gda.pl

**Keywords:** axial-flow microturbines, finite element method, fatigue stress analysis, fatigue strength, fatigue life, aluminium alloy

## Abstract

Today, where the production of any kind of device may have a negative impact on the environment, it is crucial to produce machines that are as efficient as possible but that can also be strong enough to withstand harsh operating conditions for a long time. That is why this paper raises the issue of the fatigue analysis of high-speed axial-flow microturbines whose components are made of commonly used 7075 aluminium alloy. The paper presents different methods that can be used to estimate and increase the fatigue life of a turbine disc. The object of study is a 10-kilowatt vapour microturbine. The various mechanical, flow and thermal loads that can occur during the operation of the microturbine have been analysed so that the most important ones can be taken into account in the final considerations. Stress calculations were performed using analytical equations, and the finite element method (FEM) was also used. Using the stresses obtained and material characteristics, fatigue analysis was conducted. Then, new hybrid calculation methods were proposed, taking into account both analytical and numerical approaches that do not require the use of ready-made programs dedicated to fatigue analysis. To verify these methods, calculations were performed for two rotor discs with different geometries. These methods can be used by both engineers and scientists in the design process of various microturbines when fatigue calculations are performed.

## 1. Introduction

One of the most popular topics currently discussed has been taking care of the natural environment and high-efficiency power generation based on renewable sources [1]. Its popularity is understood not only in technical terms but also in social terms [2]. Therefore, there is an emphasis on ensuring that the components of modern power machines are designed in such a way as to achieve the highest possible efficiency [3,4]. A popular solution has been to use vapour or gas turbines. In the case of these devices, a higher efficiency is often the opposite of durability, in part because one of the ways of increasing the efficiency of the device is to increase the temperature of the working fluid, which significantly increases the heat load [5,6,7]. The stresses that occur in the components of high-efficiency devices are much higher than those that occur in the components of less efficient devices. Out of concern for the natural environment, designed devices should have a long lifespan and be able to withstand many stress change cycles, thus showing great fatigue life [8,9]. This mainly concerns critical machines, that is to say, machines that have to work continuously because their operation is essential for maintaining a production process or the functioning of a power system. There is no doubt that a high-power vapour turbine operating in a coal or nuclear power plant falls into such a category [10]. A malfunction of this device may cause a voltage drop in the network, which may lead to a widespread power outage in the worst-case scenario. That is why many papers concerning stress analyses have been written, including those that describe the fatigue analyses of various vapour turbine components such as blades and casings [11,12,13]. Blade roots are particularly susceptible to damage, as shown in [14] by He et al. Alongside large power systems, distributed power generation is becoming increasingly popular, where axial-flow microturbines are increasingly being used [15,16]. These turbines are characterised by their small size and relatively simple construction. They can be easily switched on and off, which is a big advantage over the high-power vapour turbines already mentioned. The devices used for the production of electricity in distributed power generation systems are not usually seen as critical machines due to the diversification of energy production through, as its name suggests, the use of distributed energy sources. However, despite this fact, microturbines should still be both durable and reliable. It is important to promote this form of electricity production. The durability of the devices and the absence of the need for frequent maintenance inspections are undoubtedly significant advantages.

Microturbines used in distributed power generation have a significant advantage over high-power vapour turbines, as they can operate in start/stop mode, as mentioned earlier. Shutting down a power unit with an electric power greater than 1 MW is a fairly expensive process, and an unexpected shutdown resulting in a power failure can also be dangerous, and a number of measures must therefore be taken to prevent such an event from occurring [17,18]. However, the operation of microturbines under a start/stop regime also has adverse consequences, namely the appearance of fatigue stress cycles caused by changes in the rotational speed of these devices.

As shown in [19] by Kaczmarczyk et al., in microturbines used in ORC (organic Rankine cycle) systems, changes in the speed and temperature cause changes in the stresses in the rotor disc, for example, during run-ups. As mentioned earlier, microturbines can be shut down in a relatively simple manner. In terms of fatigue strength, this operating regime can be described as a from-zero pulsating cycle, that is, the turbine reaches its nominal speed and operates at that speed for some time and then is shut down. These cycles are less dangerous than symmetric cycles, which are described further in this paper. However, in spite of this, when it comes to heavily loaded discs, this needs to be considered in depth. In some cases, the from-zero pulsating cycles are particularly important, for example, for the cold starts of a steam turbine, as described by Banaszkiewicz et al. [13]. As mentioned earlier, modern devices are subjected to extremely harsh operating conditions, and in order to increase their efficiency, the nominal speed must be increased [20]. As the angular speed increases, the forces acting on the rotor disc increase, as do the stresses. Therefore, the stress change cycles associated with switching the turbine on and off are crucial.

Another important aspect is the vibration of the blades caused, for example, by secondary vortices or stator tracks. They cause vibrations that have a huge impact on the fatigue life of turbomachines [21]. Another extremely important element is the thermal stresses resulting from the operation of the turbine. They are very important during operation under high temperatures, such as those described in [22] by Sławiński et al., where the temperature reached 800 °C. Axial-flow microturbines use synchronous generators to convert kinetic energy into electric energy [23]. These are commonly used devices; however, they sometimes fail: 69% of all failures in generators can be attributed to loss-of-excitation, which causes a significant increase in speed and their instability [24]. A small number of papers on the fatigue analysis of axial-flow microturbines have been published, and few of them concern methodology for calculating such devices and the typical hazards associated with their operation. 

This paper aims to describe the main challenges regarding the fatigue analysis of axial-flow microturbines made of aluminium alloys and to define the most dangerous cyclic phenomena that can lead to their destruction. In the available literature, no one has so far paid so much attention to the fatigue analysis of a similar microturbine, although these types of machines are increasingly being used to generate electricity in small cogeneration systems. The aim of the research described in this paper was also to develop a method for calculating fatigue life for such devices based on theoretical principles and knowledge of the target operating conditions. The proposed approach takes into account all the main mechanical, aerodynamic, thermal and electrical loads. Both analytical and numerical methods were used to analyse stresses in the disc of an axial-flow microturbine. The key factors influencing the fatigue life were then defined, and the correction factors, having a significant impact on the fatigue strength, were estimated and applied. Based on the elements described above, an algorithm was developed which can be used to perform fatigue calculations of axial-flow microturbine discs made of aluminium alloys without the use of advanced models and FEM programs. In the proposed method, a fatigue analysis is carried out in two steps. In the first step, stresses are determined using an FE program. In the second step, based on the determined stresses, a fatigue analysis is performed using analytical methods for the areas where the highest stresses occur. In the proposed approach, modern numerical methods are combined with classical fatigue analysis. That is why this method has been called a hybrid method. The computational verification carried out proved the accuracy of the proposed method.

## 2. Variability of Loads and Types of Stress

**Figure 1:**
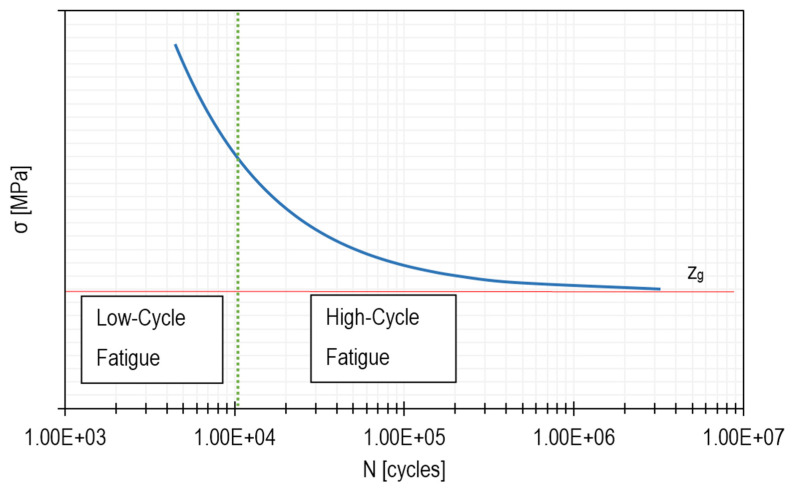
Example of a Wöhler diagram for the aluminium alloy.

Material fatigue can only be talked about when the object is subjected to variable load cycles for some time. Usually, two main types of material fatigue can be identified, namely high-cycle fatigue (HCF) and low-cycle fatigue (LCF) [25,26]. The diagram below (Figure 1) shows the division between low- and high-cycle fatigue. The limit between LCF and HCF can be set differently, but in this paper, it was adopted similarly to that in [27] by Kim and Hwang, which is about 10^4^ cycles.

The above *σ–N* diagram shows a curve that is typical of aluminium alloys, i.e., a group of materials commonly used, for example, in the aircraft industry. Such curves obtained for specific alloys are presented in the studies carried out by Zhao and Jiang [27] as well as by Yang et al. [28,29]. Thanks to the study carried out by Yang et al. [28], it was possible to create a diagram that was used for further analysis. This was done on the basis of a function that was described in detail by the aforementioned authors. The horizontal line, marked in the graph with the symbol z_g_, corresponds to the material limit. This is an asymptote of the stress value at which the sample has unlimited fatigue life. The above-mentioned diagram is called a Wöhler diagram or an *σ–N* dependence function and is created for specific materials. On its basis, it is possible to estimate the number of cycles at which material breakage occurs.

### 2.1. Load Cycles and the Equivalent Stress Amplitude

**Figure 2:**
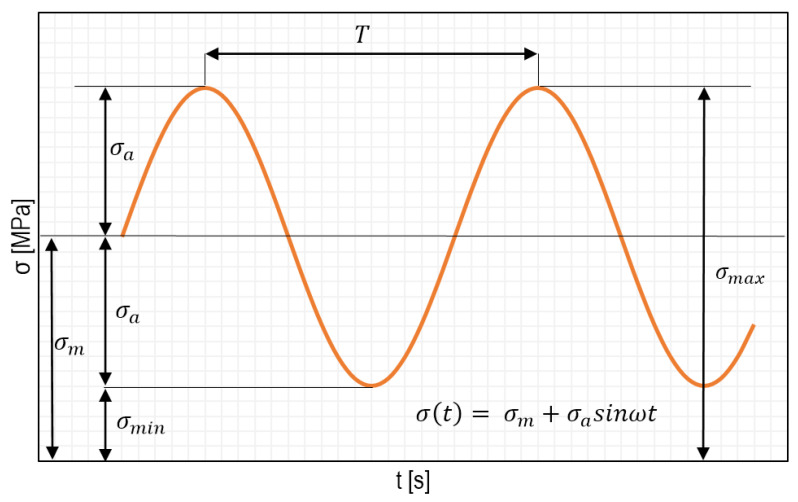
Graphical representation of the basic parameters and notations used for fatigue calculations (T stands for the load period).

To examine fatigue strength, it is necessary to become familiar with the types of stress cycles. Figure 2 shows load changes, i.e., stress as a function of time. Based on Figure 2, we can distinguish three basic cycles of stress changes (Table 1), which are defined by the following formula:

(1) $$R=\frac{\sigma_{min}}{\sigma_{max}}$$

where *R* is a stress ratio and stands for the ratio between the minimum (*σ_min_*) and maximum (*σ_max_*) stress values.

The most important variable for fatigue life is, as presented earlier, the stress amplitude (*σ_a_*) of the cycle. Its value can be determined based on analytical calculations or by using FEM.

When experimental data are not available for a given material or are provided only for conditions that occur under symmetric stress cycles (*R* = −1), the equivalent stress amplitude (*σ′_a_*) can be used to determine the number of cycles before the occurrence of a failure in the case of cyclically varying load conditions [30,31]. The equivalent stress amplitude can be calculated based on the stress amplitude (*σ_a_*), mean stress values (*σ_m_*) and parameters for a chosen material beyond which it would be destroyed [32]. This approach can be applied to a variety of materials, even to composites [33]. Several methods can be used to accurately calculate these parameters. The most commonly used are the three methods listed in Table 2, and to accurately determine the equivalent values of the stress amplitudes of the cycle, [31] by Marczewska et al. was used.

The first two methods use linear dependencies, and the third uses a parabolic dependency. The experimentally determined dependencies for plastic materials are more similar to the line determined on the basis of the Gerber method, whereas the stresses in the most dangerous notches are in good agreement with the Goodman line. These methods are used depending on which criteria (ultimate tensile strength (*R_m_*) or yield stress (*R_e_*)) are used to design the part. If yield stress is used in the calculations, which is usually the case, the most suitable method to be used is the Soderberg method.

### 2.2. Stresses Due to Centrifugal Force

A number of factors influencing fatigue life have been described earlier. It was mentioned that fatigue life is mostly affected by from-zero pulsating cycles, which result from the ON/OFF operation characteristics, i.e., switching the device on, reaching the highest speeds, running and shutting down. The highest stresses are due to rotational speed—this phenomenon is described using the radial stress ($\sigma_{rr}$) and circumferential stress ($\sigma_{\theta \theta }$) formulas given below [7]:

(2) $$\sigma_{rr}\left(r\right)=\frac{3+v}{8}\rho \omega^{2}\left[a^{2}+b^{2}-r^{2}-\frac{a^{2}b^{2}}{r^{2}}\right]\;$$

(3) $$\sigma_{\theta \theta }\left(r\right)=\frac{3+v}{8}\rho \omega^{2}\left[a^{2}+b^{2}-\frac{1+3v}{3+v}r^{2}+\frac{a^{2}b^{2}}{r^{2}}\right]$$

**Figure 3:**
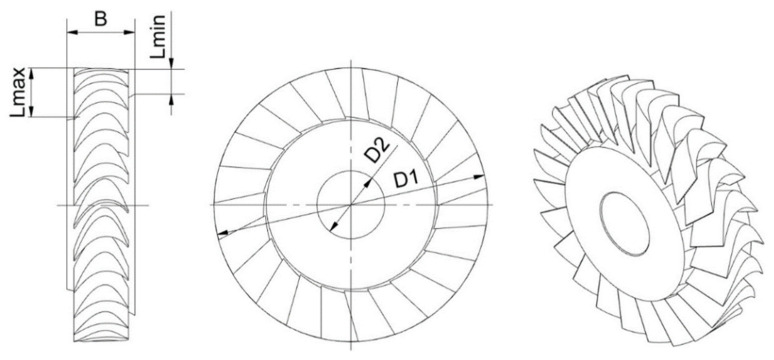
Geometry and basic dimensions of the rotor disc of a 10 kW axial-flow microturbine.

The above stresses refer to breaking the disc into pieces due to rotations, where b stands for the inner diameter (which corresponds to *D*2 in Figure 3) and *r* stands for the radius used for stress calculations. As far as the inner diameter value is concerned, in order to get as close as possible to the dimensions of the rotor disc, a decision was made that the value of the diameter a would be equal to the mean value referring to the average blade height and calculated as below (Equation (4)), where *D*1 stands for the outer diameter (see Figure 3) and *L_a_* stands for the average blade height. Since the filling of the blade space with the material in this case is about 50%, the outer diameter of the bladed disc was reduced by the average blade height. In this way, the mass of the blades has been taken into account in the disc calculations.

(4) $$a=D1-L_{a}$$

The von Mises hypothesis was used to obtain reduced stresses based on radial and circumferential stresses.

(5) $$\sigma_{red}=\sqrt{\sigma_{rr}{}^{2}-\sigma_{rr}\sigma_{\theta \theta }+\sigma_{\theta \theta }{}^{2}\;}$$

On the analytical level, the issue was simplified, and only a flat stress state has been considered in the further part of this paper.

### 2.3. Stresses Resulting from the Irregular Supply

Another aspect that can affect the fatigue life is the irregular supply to the turbine, which causes the forces acting on the blades to change over time due to the movement of the rotor in relation to the stator and also due to the clearance between the blades. This is a non-stationary flow of the fluid flowing through the blade cascade, and its magnitude can be determined by the following formula:

(6) $$\kappa_{w1}=\kappa_{c1}\frac{1-\frac{u}{c_{1}}\cos\alpha }{{\left(\frac{u}{c_{1}}\right)}^{2}-2\frac{u}{c_{1}}\cos\alpha +1}$$

where $K_{w1}$ is the fluid flow magnitude coefficient, $K_{c1}$ is the fluid flow field irregularity coefficient, *u* is the peripheral velocity, $c_{1}$ is the average fluid velocity and α is the inlet angle. 

Changes in the influx to the rotor come from so-called edge tracks. They cause resonance vibrations of the blades that, in turn, cause dynamic stresses in each blade, which can be calculated by multiplying the reduced (von Mises) stresses by the corresponding coefficients so that a symmetric stress cycle can be considered in this case, where:

(7) $$\sigma_{dyn}=\sigma_{red}\cdot C_{i}\cdot A_{j}$$

(8) $$\sigma_{dyn}=\sigma_{a}$$

In the above formula, $C_{i}$ stands for the coefficient of the ith harmonic, and A stands for resonance amplitude of the *j*th order. The resonance phenomenon can occur when the vibrations of the blades are not dampened and when the harmonic frequency (*ω_i_*) is equal to any natural frequency of the blade (*υ_j_*). When a blade is treated as a bar, the natural frequency can be expressed by the following formula:

(9) $$\vartheta_{j}=k_{j}\sqrt{\frac{EI}{\rho_{m}Al_{max}{}^{4}}}$$

In high-power turbines, natural frequencies and their multiples have a huge impact on dynamic stresses and, consequently, on fatigue life [34]. This is due to the fact that the coefficient *A_j_* takes the highest value for the first natural frequency. As the above formula shows, in the case of small turbines where the blades are very short and, therefore, the surface area of the blades is small, the natural frequencies will be very high, and none of them will be approximately equal to the nominal speed. This phenomenon has been perfectly described by Żywica et al. in [35], where at a rotor rotational frequency of 397 Hz, the natural frequency was as high as 90,660 Hz. It can therefore be concluded that, although vibrations are an extremely important factor when analysing the fatigue life of turbomachines, they do not play such an important role in the case of axial-flow microturbines.

### 2.4. Other Variable Stresses

The aforementioned instabilities may also result from the loss of synchronism of the rotor of the generator and, consequently, of the entire rotating system, which leads to fluctuations in the rotational speed, creating a symmetric cycle [36]. In this case, there is no electromagnetic braking torque acting on the rotor of the generator, which can lead to uncontrolled changes in speed. Such a situation is very dangerous, as it can lead to the destruction of the entire rotating machine.

The temperature variation over time during operation is extremely important. Turbines used in ORC systems, as described in [19] by Kaczmarczyk et al., show significant variations of this parameter, for example, during start-ups. Thermal resistance, and consequently thermal durability, is a very complex issue, as shown, for example, in [22] by Sławiński et al. Analyses of these types of stresses can be extremely extensive and complex, which is why the authors of this paper focused solely on mechanical loads. However, the effect of temperature itself has been taken into account in the following part of this paper in the form of a correction factor that takes into account the deterioration of the mechanical properties of the aluminium alloy with increasing temperature. Our analyses have shown that, in the case of the vapour microturbine, whose rotor disc is made of an aluminium alloy and has small dimensions, such mapping of the temperature effect is sufficient. Aluminium alloys have very good thermal conductivity, which, given the small size of the disc, promotes the appearance of a uniform temperature distribution. Under these conditions, there is no large temperature gradient within the rotor disc, so no large thermal stresses are generated either.

### 2.5. Object of Study

**Figure 4:**
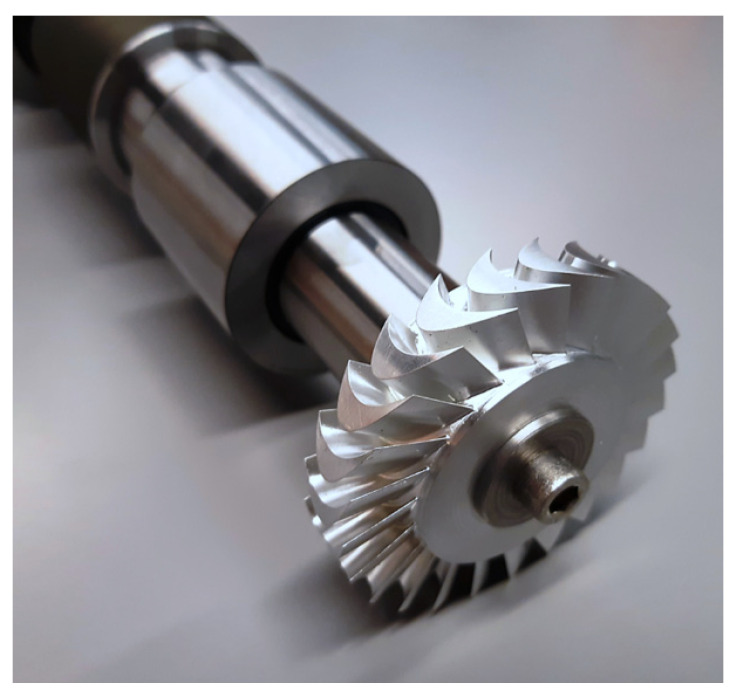
Photograph of the real rotor with the turbine disc that was subjected to analysis.

In this paper, analytical calculations were performed on axial-flow turbine discs using one such component from a 10 kW vapour microturbine, and then calculations were performed using the FEM method. The flow optimization of a similar turbine disc is described by Witanowski et al. in [37]. A photograph of a real rotor is shown in Figure 4.

To start dealing with variable stress cycles, one has to begin first with stresses. This paper focuses exclusively on the discs of axial-flow microturbines and on the phenomena that occur in these structures. Let us consider the disc of the axial-flow microturbine, which has been presented earlier and whose dimensions are presented in Figure 3 and described in Table 3.

The disc is made of aluminium alloy 7075 (PA9), often used in the power engineering and aircraft industry. Aluminium alloy 7075 is composed of 90% Al, 5.6% Zn, 2.5% Mg, 1.6% Cu and 0.23% Cr [38]. The mechanical properties of this material are presented in Table 4.

The next step was to determine possible cycles of stress changes, based on which fatigue calculations were performed both analytically and with FEM. In addition, thanks to the CFD calculations, the rotations of the turbine were taken into account, which cause changes in stresses over time resulting from the leaning of the blade palisade away from the stator. An algorithm using analytical methods and FEM was developed. In addition, several fatigue hypotheses were used, and the results thus obtained were compared.

## 3. Results

### 3.1. Calculations of Stresses Using the Analytical Method

The MatLab program was used to calculate the reduced stress distribution in the entire disc at a rotational speed of 50,000 rpm. To perform the calculations during the first analytical stage, in order to estimate the stress values in individual parts, the disc was “divided” into a disc with a hole and blades. During analytical calculations, the stresses in these parts were determined independently and were not added together.

**Figure 5:**
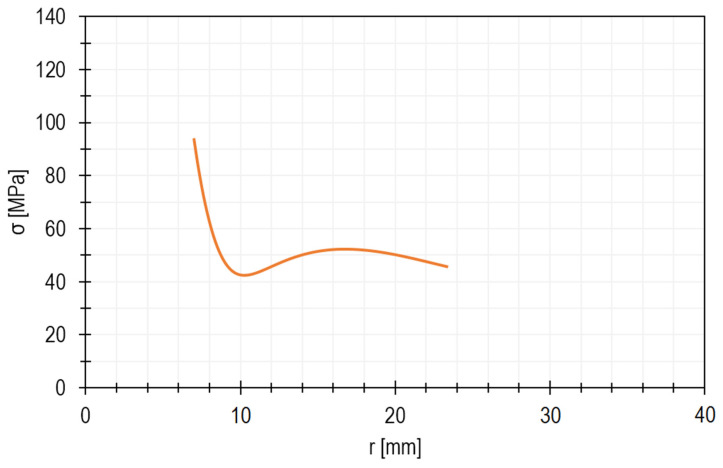
Stress distribution in the microturbine disc depending on the radius.

As shown in Figure 5, which illustrates the stress distribution in the disc, the highest reduced stress is 92 MPa and occurs where the radius is the smallest, that is, at the edge of the hole. As mentioned before, the blades were not taken into account in these calculations, so the calculated value can be greater than that within the hole. This simplified analysis made it possible to estimate the place where the axial-flow microturbine disc is expected to be subjected to the greatest stresses.

The next step is the calculation of the blade itself because this is where the complex state of stresses acts. First of all, the stresses that can rupture the blade are the greatest, and they increase with rotational speed. They can be calculated using the following formula:

(10) $$\sigma_{rl}=\frac{1}{2}\rho L_{max}D_{1}\omega^{2}$$

The stress value calculated according to the above formula is 58.4 MPa. The greatest stress in the blade occurs at its junction with the disc (i.e., in the foot of the blade). This value may be overestimated because the blade does not have a constant height. However, the differences should not be significant. Therefore, it can be seen that the highest stresses occur near the microturbine disc hole.

### 3.2. Stress Calculations Using FEM

In order to perform FEM calculations, a discretized three-dimensional model of the disc was created using the ANSYS software [40]. The number and size of the finite elements have been selected so as to obtain accurate results in the shortest possible time. A dense FEM mesh was used in the locations where geometric discontinuities occur to properly represent stress concentrations. The correct representation of stress concentrations in irregular components made of aluminium alloys is very important for the proper prediction of locations where crack propagation may occur [41]. The fatigue life of these components can also be strongly affected by surface porosity [42]. However, this aspect was not taken into account in our analyses due to the very fine machining of the microturbine components and the limitations of the FEM model. Finally, the FEM model consisted of 1,417,547 elements and 2,389,164 nodes. The FEM mesh, which included such a large number of elements and nodes, very accurately represented the three-dimensional geometry of the rotor disc of the microturbine and the geometric discontinuities occurring at the junction of the blades with the disc. 

**Figure 6:**
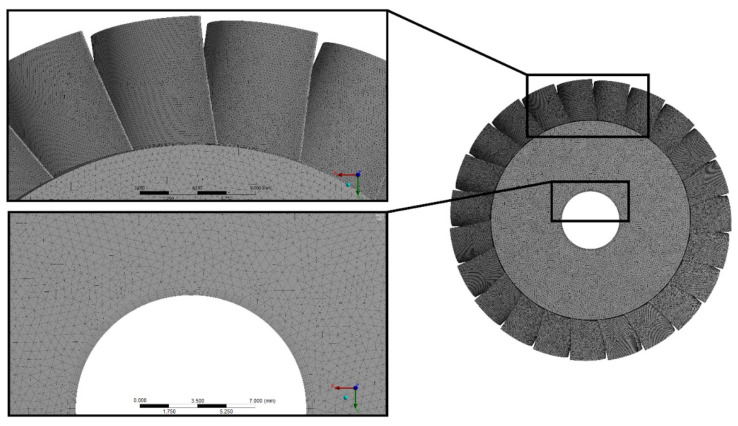
Discretized model of a microturbine rotor disc.

When analysing the strength of turbine discs, the symmetry condition called “cyclic region” is usually applied, which allows a blade to be discretized and duplicated so many times until the whole disc is created. During fatigue calculations that will be carried out at a later stage, this condition is not applied, as the model must be homogeneous. Due to the complexity of the geometry, the mesh is tetragonal and is built from prismatic elements. In addition, a boundary layer has been created in areas where stresses were expected to be the greatest, i.e., at the edges of the hole. The mesh is of good quality in terms of orthogonality. The concept of mesh orthogonality relates to how close the angles between adjacent element faces or adjacent element edges are to some optimal angle (depending on the relevant topology). In areas where the stresses are the highest, the value of this parameter is 0.9. Figure 6 shows the discrete model.

**Figure 7:**
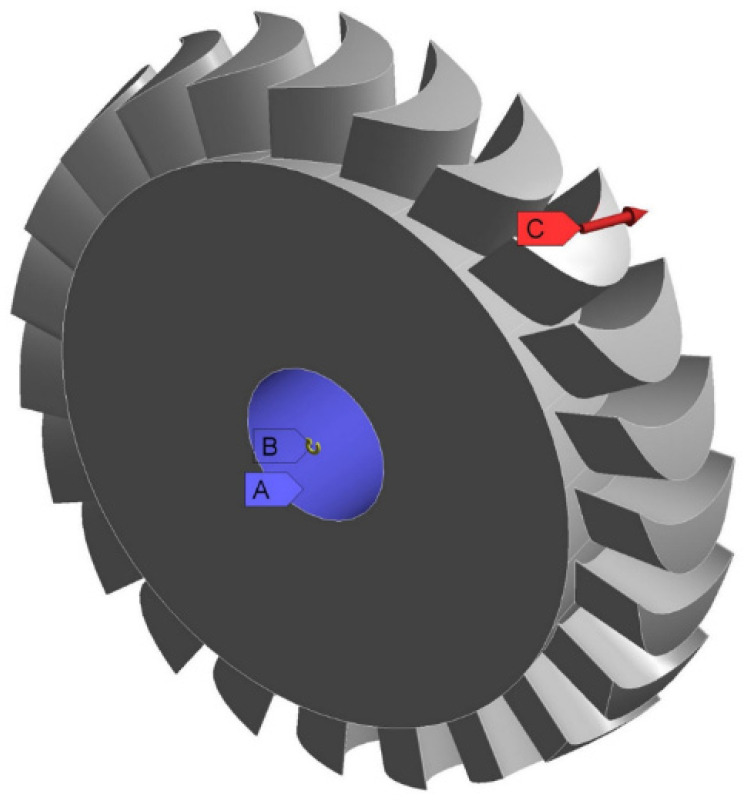
Boundary conditions applied to the calculations of microturbine disc.

During analytical calculations, the entire disc was simplified and divided into two elements—the disc and the blade—which were analysed separately. FEM makes it possible to calculate the whole object, taking into account the influence of the blades on the disc and vice versa [43]. It is therefore necessary to estimate the maximum force acting on the blade ring, which results from the fluid flow. This force changes over time because the microturbine disc rotates. This is discussed in more detail in the later part of this paper. In order to determine the value of the force acting on the blades, calculations were performed using CFD software. The boundary conditions used in the calculations are listed in Table 5 and shown in Figure 7. The cylindrical support (A) applied over the entire surface of the central hole of the disc means that displacements in the radial and axial directions are blocked. Condition B means that the rotational speed—which causes the appearance of a centrifugal force in the disc—is set with respect to the point located on the main axis of the disc. Condition C symbolises the loading of a single blade by the force resulting from the flow of the working fluid through the microturbine. During the analysis, the same force also acts on the other blades of the rotor disc.

**Figure 8:**
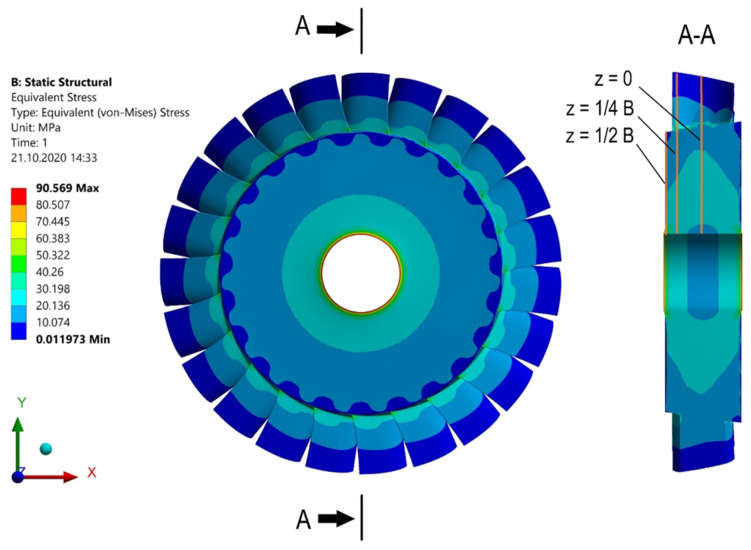
Stresses in the microturbine disc for a speed of 50,000 rpm.

**Figure 9:**
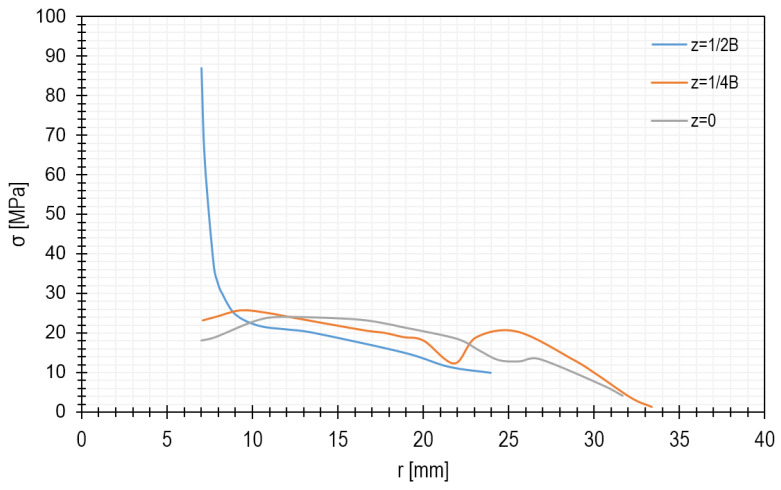
Stress distribution in the microturbine disc along the radius.

Figure 8 shows the stress distribution. As seen, the highest stresses occur at the outer edge (z = ½ B), near the hole. The stress as a function of the radius of the microturbine disc can be seen more precisely in the diagram shown in Figure 9. In general, it can be stated that the reduced stresses decrease with increasing distance from the centre of the disc and vary along its thickness.

### 3.3. Fatigue Analysis

Table 6 describes four load states that can occur under normal operating conditions. The first mode (C1) indicates switching the turbine on and off and is a from-zero pulsating cycle where the stresses increase from zero to the maximum value resulting from the nominal speed of the rotor. The next mode (C2) indicates the irregular supply caused by the stator track. As shown earlier, the turbine disc eigenmodes occur at much higher speeds than the nominal speed (50,000 rpm); however, as the CFD calculations have shown, the forces acting on the blades are not constant. The value of the stress amplitude (*σ_a_* = 0.5 MPa) was taken from the FEM calculations and was equal to the highest value of reduced stresses occurring in the blade, taking into account the pressure distribution calculated in the CFD program. The highest stresses due to the flow of the working fluid occurred at the foot of the blade on the supply side. However, these stresses were much lower than those caused by the centrifugal force (even at nominal speed). C3 corresponds to the loss of excitation of the synchronous generator. This is a situation where the braking torque ceases to act in the generator, resulting in an uncontrolled and very rapid change in rotor speed. Based on the previously mentioned papers, the speed changes were classified as a symmetric cycle, where speed increases to 1.4 *n* and decreases to 0.6 *n*, that is, oscillates around the nominal speed (plus/minus 40%). This is an extremely dangerous case that can lead to damage to the rotor and the entire microturbine. The last case (C4) is an emergency that indicates the temporary loss of generator power, where *n_2_* = 1.4 *n*. This case is also in line with the previously mentioned papers and concerns the quick reaction of the system to the loss of load of the generator, that is, an increase in speed above the designed speed, then a return to the nominal speed.

Based on the calculations presented previously, maximum stress values were obtained using both analytical ($\sigma_{maxA}$) and numerical ($\sigma_{maxN})$ methods, and these values are presented below:

$$\sigma_{maxAC1}=92\;\mathrm{MPa};\;\sigma_{maxNC1}=90\;\mathrm{MPa}$$

$$\sigma_{maxAC3}=184\;\;\mathrm{MPa};\;\sigma_{maxNC3}=177\;\mathrm{MPa}$$

$$\sigma_{maxAC4}=184\;\mathrm{MPa};\;\sigma_{maxNC4}=177\;\mathrm{MPa}$$

In order to accurately reflect the reality, a fatigue strength coefficient (*K_f_*) has been defined, which reduces the fatigue limit value depending on a number of variables described below [30,44]. The overall fatigue strength coefficient is determined by multiplying the individual coefficients presented in Table 7.

(11) $$K_{f}=k_{a}\cdot k_{s}\cdot k_{t}\cdot k_{l}$$

These coefficients reflect various features of the microturbine disc (e.g., surface quality and irregular shape) and the operating conditions (e.g., elevated temperature and loads). Their recommended values and guidelines for their selection can be found in scientific papers or handbooks [30,44,45]. In our case, although the central hole was taken into account in the disc models, the shape influence coefficient was assumed to be around 0.7. This is because the actual rotor disc has two additional smaller holes located near the central hole, which serve to secure the pins allowing the torque to be transferred from the disc to the shaft. Additional FEM analyses showed that these smaller holes significantly increase the stresses near the central hole. Therefore, in the simplified models used for fatigue analysis, this was represented by a correction factor (*k_s_*) equal to 0.7. Similar effects can be expected with a keyway groove, which is quite commonly used. The same values of fatigue strength coefficients were used for all analyses discussed later in this paper.

After taking into account the individual coefficients given in Table 7, the overall fatigue strength coefficient, rounded to one decimal place, was 0.2. After the value of the coefficient *K_f_* was set, the equivalent stresses were applied, taking into account the previously described elements, which change the values in the *σ–N* diagram, where:

(12) $${\sigma_{a}}^{\prime }=\sigma_{a}/K_{f}$$

The *σ–N* diagram, which is necessary to perform the calculations, was created on the basis of the correlation described below [46]:

(13) $$\log N=18.21-7.73\cdot \log\left(\sigma_{f}-10\right)$$

(14) $$\sigma_{f}=\sigma_{max}{\left(1-R\right)}^{0.62}$$

**Figure 10:**
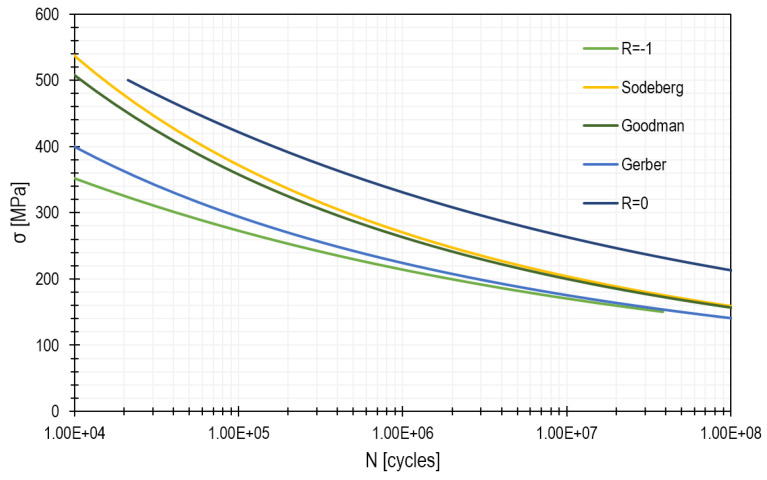
*σ–N* graph for aluminium alloy 7075 with reduced stress values.

Therefore, in the case under examination, for the from-zero pulsating cycle, *R* = 0. Figure 10 shows the diagrams created on the basis of the function described above. In addition, three diagrams of the reduced functions were plotted in relation to cycle *R* = −1.

The biggest difference occurs when the Gerber method is applied. However, as mentioned earlier, if the calculations refer to the yield stress Re, the Soderberg method is the most appropriate one, and at the same time, it corresponds most closely to the material data available for *R* = 0. Reductions were made to indicate that their applicability is possible in case of lack of data; however, for aluminium alloy 7075, it is possible to refer directly to the *σ–N* diagram for the from-zero pulsating cycle.

### 3.4. Proposed Calculation Method

**Figure 11:**
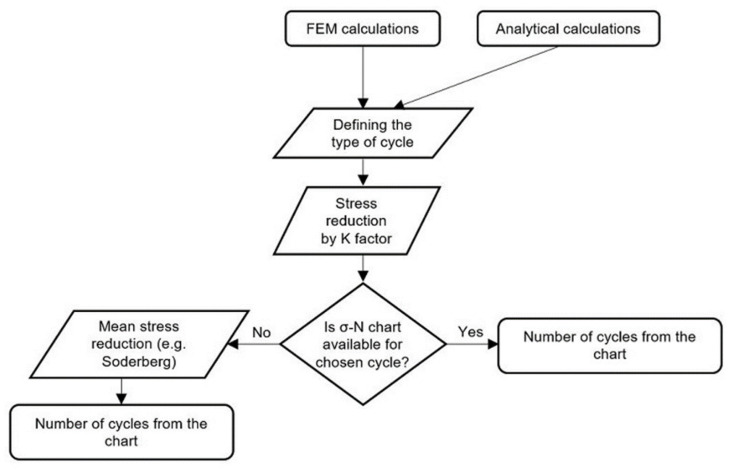
Block diagram of the fatigue calculations performed using the proposed method.

When the maximum stress value ($\sigma_{maxA}$) has been calculated analytically, the type of cycle and the amplitude stress ($\sigma_{A}$) must be determined and then reduced using the fatigue strength coefficient (*K_f_*). If the material data are available, that is to say, the *σ–N* diagram for the selected load cycle is at our disposal, the amplitude stress values obtained should be compared to those shown in the diagram. If such data are not available, the Soderberg reduction method should be used. Another proposed method is the one that uses numerical calculations to obtain the maximum stress value ($\sigma_{maxH}$), and further calculations should be carried out in the same way as for the analytical method. The proposed algorithm is shown below as a block diagram (Figure 11).

In the method we have proposed, the fatigue analysis is therefore carried out in two steps. In the first step of the analysis, any FEM program capable of performing strength analyses can be used to determine the stresses. In the second step, on the basis of the previously determined stresses, a fatigue analysis is performed using analytical methods for selected areas of the structure where the highest stresses occur. Since during these calculations modern numerical methods are combined with classical fatigue analysis, the proposed approach has been termed a hybrid method.

**Figure 12:**
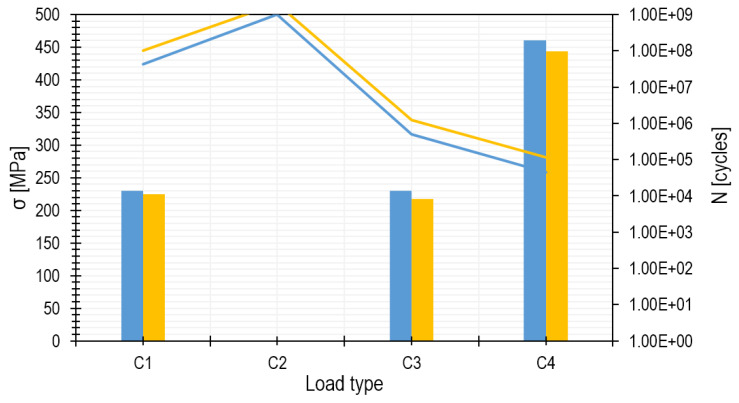
Results of calculations performed using the analytical (blue colour) and hybrid (orange colour) methods.

The number of operating cycles for both analytical and numerical methods and the load conditions presented in Table 6 were determined, and the results are shown in Table 8 and Figure 12. The values of the equivalent stress amplitudes were determined using the formulas given in Table 6 on the basis of the previously determined stresses, taking into account the fatigue strength coefficient (according to Equation (12)). The different numbers of cycles obtained for similar values of equivalent stress amplitudes using different load scenarios are due to the reference to different *σ–N* curves (Figure 10), depending on the characteristics of the load.

In Figure 12, the bars indicate the stress values, and the curves represent the number of cycles. If one takes into account the completely different approaches of various methods, the obtained stress differences should be considered small. For engineering calculations, the analytical method is safe, as the number of cycles is understated. As far as accuracy is concerned, the hybrid method that uses three-dimensional geometry and FEM calculations is much more accurate, as it uses a three-dimensional geometry of the system and takes into account all geometric discontinuities. The analytical method takes into account only the flat stress state determined by the simplified model. If there were any technological or structural elements such as an undercut, phase or pinhole, the analytical method might have not been suitable, as it does not take into account stress concentrations. This has been described quite precisely in [43] by Bagiński et al., where the authors dealt with the discretization of a calculation model.

### 3.5. Verification of the Proposed Method

To verify the results obtained, additional calculations were carried out using ANSYS Workbench. In this program, universal ready-made tools for fatigue calculations are available. In the ANSYS Fatigue Module, two methods (Strain Life and Stress Life) are available for low- and high-cycle fatigue analysis. An analysis can be performed using four different load types: fully reversed, zero-based, ratio and history data. Since actual components seldom experience a pure type of loading as a fully reversed one, this module allows us to use the mean stress correction. The calculation method and the results are dependent upon the type of fatigue analysis chosen. As a result of calculations, we can obtain contour plots of specific parameters such as fatigue life, fatigue damage at a specified design life, fatigue safety factor at a specified design life or fatigue sensitivity charts, among others [40]. This software can be used to perform fatigue analysis of different machine components such as notched cantilever beams [47], engine connecting rods [48] or tubular welded assemblies [49]. This program also successfully passed the experimental verification, which was carried out for the notched specimen [50]. The predicted results agreed well and consistently with experimental data. This program can therefore be regarded as a reliable calculation tool. 

**Figure 13:**
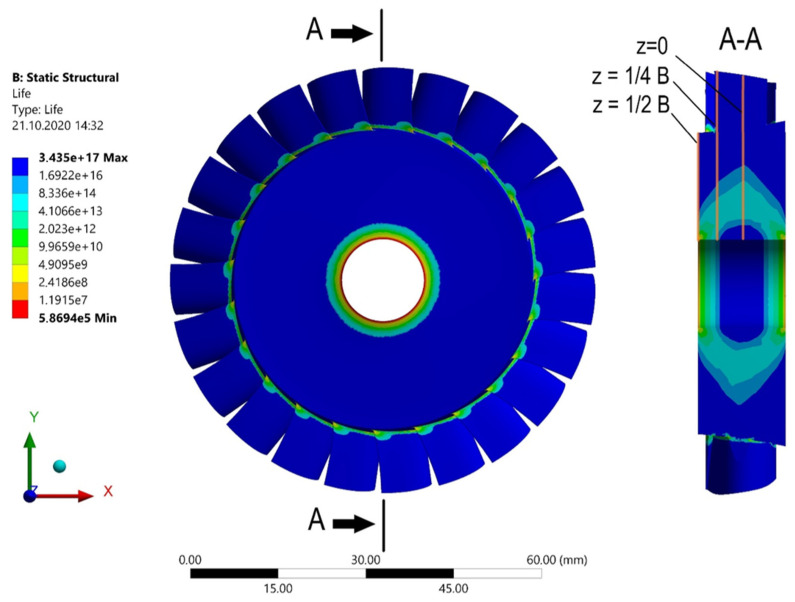
Life prediction for microturbine disc at a speed of 50,000 rpm (C1 case).

Due to the limited number of experimental results that can be used to validate numerical models used for fatigue analysis, the authors of some papers validate their models by making direct reference to results obtained using the ANSYS Workbench program [51]. A similar approach has been proposed in this paper. The Stress Life model was applied for the analysis, taking into account mean stress effects and a fatigue strength coefficient. The same loads and boundary conditions as those used for the previous strength calculations were applied. The results of the fatigue analysis are shown in Figure 13.

**Figure 14:**
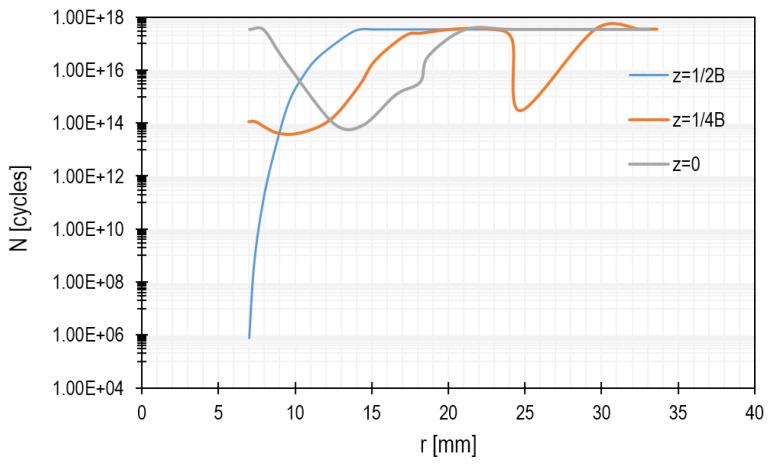
Cycles versus radius of the microturbine disc for different cross-sections of the disc.

The highest stresses occur near the hole, so the number of operation cycles will be the lowest there. The diagram included in Figure 14 shows the exact distribution of the number of cycles along the lines presented in Figure 13. These curves were determined using the ANSYS Workbench program and correspond to the maximum number of cycles resulting from the stresses determined in the cross-sections of the microturbine disc (at different distances from the middle plane).

Comparing the results, it can be seen that they are very similar to those obtained using the hybrid method. This is due to the fact that the material data are the same in both cases. Small differences result from matching the values from the *σ–N* diagram to the results obtained in the ANSYS Workbench program. This program uses logarithmic or linear interpolations. The linear method was chosen because the results were the most consistent with those determined on the basis of the *σ–N* dependence for aluminium alloy 7075.

### 3.6. Calculations Performed for a Disc with a Different Diameter

As the dimensions of the microturbines increase, the stresses also increase; moreover, the stresses also increase with the increase of the *D1/Lmax* quotient. The highest stresses can then occur in the blades themselves. In the case analysed, this ratio is 0.4, and a similar situation occurs in most ORC microturbines. However, if the overall dimensions of the turbine increase, the stresses also increase, and, consequently, the number of cycles decreases significantly. To support this thesis, the fatigue life was calculated using the analytical method and the hybrid method for the turbine whose dimensions are described in Table 9.

**Figure 15:**
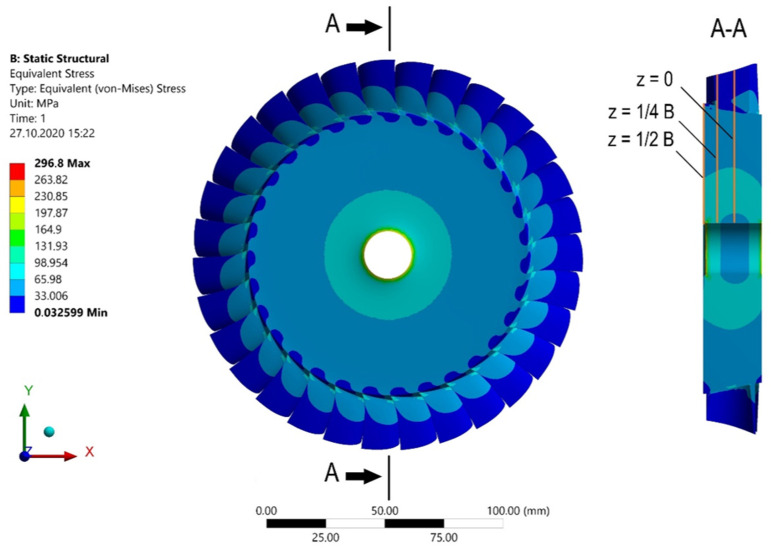
Stress distribution obtained for the microturbine with an outer diameter of 146 mm at a speed of 40,000 rpm.

**Figure 16:**
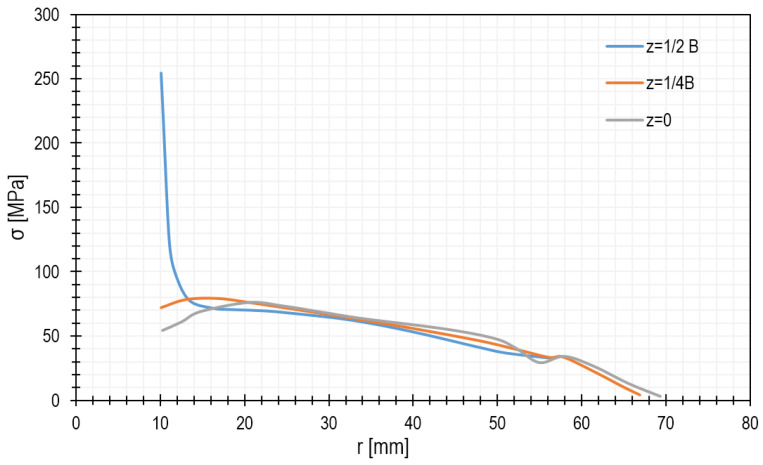
Stress distribution depending on the radius of the microturbine disc with an outer diameter of 146 mm at a speed of 40,000 rpm.

The rotational speed of the turbine is 40,000 rpm, and it is made of aluminium alloy 7075. The analytically calculated reduced stresses are 125 MPa on the blades and 284 MPa near the edge of the hole. The results of the numerical calculations are shown in Figure 15 and Figure 16.

For the from-zero pulsating cycle, where *σ_A_* = 142 MPa and the coefficient *K_f_* was equal to 0.20, the number of cycles that would cause the destruction of the turbine disc was determined. The number of cycles, which, in this case, leads to destruction, is approximately 850. These results clearly show that the dimensions and geometric proportions of the rotor disc have a considerable impact on the fatigue life. 

## 4. Conclusions

The purpose of this paper was to determine the basic load variations in rotor discs of axial-flow microturbines and to assess their impact on the fatigue life. Several examples of loads that can have a significant impact on the system have been presented. Emergencies resulting from excitation loss have proved to be the most dangerous. However, it is still a high-cycle strength. As shown in the paper, axial-flow microturbines are not very sensitive to high-cycle strength.

Since one of the most important advantages of micro energy systems is their ability to operate in ON/OFF mode, it has been proved that the most important element of the system can withstand more than 4.2 × 10^7^ of these types of cycles. This makes it possible to conclude that it is a very durable construction. Interestingly, the irregular supply to the microturbine does not have a significant impact due to the fact that the working fluid is a low-boiling substance and therefore has a fairly low density, so the forces it exerts on the blades are not great.

The proposed analytical calculation method allows the number of cycles to be estimated on the basis of the disc dimensions and available material data. Not every commercial FEM program has the availability to make fatigue analysis and to calculate a number of cycles, which is why hybrid and analytical methods described in the paper could be implemented in a design process in a simple way, especially while designing high-speed microturbines made of aluminium alloys. What is more, it could be used easily after performing regular strength calculations during the design process. The fatigue life of a microturbine is a very important aspect of the operation of the entire energy system; therefore, such calculations are really important during the design process of microturbines, especially if the *D/Lmax* ratio is greater than 0.4. The next stage of work will be to evaluate changes of the rotational speed of a real rotating system in terms of load variations and to propose a calculation method that would take into account the loads of the axial-flow microturbine that occur under operational conditions.

## Figures and Tables

**Table 1:** Conditions for different stress cycles.

Type of Cycles	Conditions
Constant stresses	$\sigma_{min}=\sigma_{max}=\sigma_{m},\;\sigma_{a}=0,\;R=1\;$
Positive from-zero pulsating cycle	$\sigma_{max}>0,\;\sigma_{min}=0,\;\sigma_{m}=\sigma_{a}=\frac{1}{2}\sigma_{max},\;R=0\;\;$
Oscillatory cycle, symmetric	$\sigma_{max}=-\sigma_{min}>0,\;\sigma_{m}=0,\;\sigma_{a}=\sigma_{max},\;R=-1$

**Table 2:** Methods for calculating the equivalent stress amplitude.

Method	Correlation
Goodman method	$\sigma_{a\;R\neq -1}^{\prime }=\sigma_{aR=-1}\left[1-\left(\frac{\sigma_{m}}{R_{m}}\right)\right]$
Soderberg method	$\sigma_{a\;R\neq -1}^{\prime }=\sigma_{aR=-1}\left[1-\left(\frac{\sigma_{m}}{R_{e}}\right)\right]$
Gerber method	$\sigma_{a\;R\neq -1}^{\prime }=\sigma_{aR=-1}\left[1-{\left(\frac{\sigma_{m}}{R_{m}}\right)}^{2}\right]$

**Table 3:** Geometric data of the rotor disc.

Parameter	Value
*D1*—outer diameter	65 mm
*D2*—diameter of the disc hole	16 mm
*B*—width	16 mm
*Lmax*—maximum blade height	12 mm
*Lmin*—minimum blade height	6 mm

**Table 4:** Mechanical properties of aluminium alloy 7075 based on [39].

Temperature	Density	Tensile Yield Strength	Ultimate Tensile Strength	Young’s Modulus	Poisson Ratio	Fatigue Strength
22 °C	2.81 kg/m^3^	503 MPa	572 MPa	71.7 GPa	0.33	159 MPa

**Table 5:** Specification of the boundary conditions that are presented in Figure 7.

Label	Name	Value
A	Cylindrical support	x = 0 mm y = 0 mm z = 0 mm
B	Rotational speed	50,000 rpm
C	Force acting on the blade, which results from the gas flow	9 N

**Table 6:** Description of operation cycles.

Notation	Description	Cycle Type	Calculation Assumptions
C1	ON/OFF mode	From-zero pulsating cycle	$\sigma_{a}=\frac{\sigma_{maxC1}}{2}$
C2	Irregular supply	From-zero pulsating cycle only for loads of the non-rotating blades	$\sigma_{a}=0.5\;\mathrm{MPa}$
C3	Unstable system operation—falling out from synchronism	Symmetric cycle	$n_{C3}=0.6n÷1.4n\;\sigma_{a}=\frac{\sigma_{maxC3}-\sigma_{maxC1}}{2}$
C4	Emergency situation	Pulsating cycle	$\sigma_{a}=\frac{\sigma_{maxC4}}{2}\;\sigma_{maxC4}\;for\;n_{2}=1.4n$

**Table 7:** Individual coefficients adopted on the basis of [44].

Coefficient	Description	Value
*k_a_*	Surface quality—machining, without polishing	0.85
*k_s_*	Element shape—disc with a hole	0.7
*k_t_*	Effect of temperature—170 °C	0.35
*k_l_*	Load type—rotating disc with a hole	0.9

**Table 8:** Results of fatigue calculations.

Notation	${\sigma_{aA}}^{’}$	${\sigma_{aH}}^{’}$	N_A_ (Cycles)	N_H_ (Cycles)	Percentage Difference
C1	230 MPa	225 MPa	42,861,116	58,694,159	7%
C2	0.5 MPa	0.5 MPa	unlimited	unlimited	-
C3	230 MPa	217.5 MPa	492,674	756,227	34%
C4	460 MPa	442.5 MPa	45,072	67,632	33%

**Table 9:** Dimensions of the rotor disc of an axial-flow microturbine.

Parameter	Value
*D1*—outer diameter	146 mm
*D2*—diameter of the disc hole	20 mm
*B*—width	16 mm
*Lmax*—maximum height of the blade	23 mm

## Data Availability

Data are contained within the article.
